# Evidence That Nine Autistic Women Out of Ten Have Been Victims of Sexual Violence

**DOI:** 10.3389/fnbeh.2022.852203

**Published:** 2022-04-26

**Authors:** Fabienne Cazalis, Elisabeth Reyes, Séverine Leduc, David Gourion

**Affiliations:** ^1^Centre d’Analyse et de Mathématique Sociales, CNRS-EHESS, Paris, France; ^2^Auticonsult, Paris, France; ^3^Independent Practitioner, Paris, France; ^4^GHU Paris Psychiatrie & Neurosciences, Hôpital Sainte Anne, Paris, France

**Keywords:** autism, women, sexual violence, revictimization, post traumatic stress disorder, underage, abuse

## Abstract

**Background:**

Research indicates that sexual violence affects about 30% of women in the general population and between two to three times as much for autistic women.

**Materials and Methods:**

We investigated prevalence of sexual abuse, autistic traits and a range of symptoms, using an online survey addressed to the women of the French autistic community (*n* = 225). We assessed victimization through an open question and through a specific questionnaire, derived from the Sexual Experiences Survey-Short Form Victimization.

**Results:**

Both case identification methods yielded high figures: 68.9% victimization (open question) compared to 88.4% (standardized questionnaire). Two thirds of the victims were very young when they were first assaulted: among 199 victims, 135 were aged 18 or below and 112 participants were aged 15 or below. 75% of participants included in our study reported several aggressions. Analyses indicate that primo-victimization was highly correlated to revictimization and that being young increased that risk. Young victims were also at higher risk of developing post-traumatic stress disorder. A third of the victims reported the assault. 25% of those were able to file a complaint (*n* = 12) and/or receive care (*n* = 13). For the remainder 75%, reporting did not lead to action.

**Discussion:**

Those findings indicate a very large proportion of victims of sexual assault among autistic women, consistently with previous research. The World Health Organization states unambiguously that sexual violence is systemic and that vulnerable individuals are preferably targeted by offenders. We therefore postulate that it would be erroneous to consider that victimization of autistic women is mainly due to autism. On the contrary, autism seems to be just a vulnerability factor. Some authors propose that educating potential victims to better protect themselves would help preventing abuse. We reviewed this proposition in the light of our results and found it to be impossible to apply since more than half of the victims were below or at the age of consent. Literature about sexual violence is discussed. Large-scale prevention programs proposed by World Health Organization and the Center for Disease Control aim at cultural changes in order to diminish gender inequality, that they identify as the very root of sexual violence.

## Introduction

Sexual violence is a major societal issue. World Health Organization (WHO) states unambiguously that ‘‘Sexual violence is a serious public health and human rights problem with both short- and long-term consequences on women’s^1^ physical, mental, and sexual and reproductive health. Whether sexual violence occurs in the context of an intimate partnership, within the larger family or community structure, or during times of conflict, it is a deeply violating and painful experience for the survivor.’’^2^ Such wide-ranging consequences include long-term depression, post-traumatic stress disorder (PTSD), substance abuse and suicide (Devries et al., 2011; Hailes et al., 2019). It is well demonstrated that women and vulnerable individuals (children, disabled persons, elderly…) are at higher risk of victimization (WHO, 2002; Machisa et al., 2017; Willott et al., 2020). For example, prevalence of sexual abuse in adults with intellectual disability reaches 32.9% (Tomsa et al., 2021). By an intersecting effect, women with cognitive disabilities, such as autism or attention deficit hyperactivity disorder, are therefore at increased risk of being sexually assaulted. In this study, we have investigated rates of sexual assault among a group of women on the autistic spectrum, expecting elevated levels of victimization.

### Proportion of Victims of Sexual Abuse in the General Population

Sexual victimization is very common in the general population (Sardinha et al., 2022). However, it has long been under-recognized (Leight, 2022) because victims remain silent, due to limitations in being able to report and reports often not being believed. This is where sexual abuse differs from other forms of violence: most victims who report it are met with suspicion and denial and therefore are reluctant to talk about it (Ahrens, 2006; Suarez and Gadalla, 2010; Kennedy and Prock, 2018). While the underlying causes of such negative responses remain to be fully elucidated, they can be explained by the strong persistence of myths about sexual violence (e.g., “women provoke rape by the way they dress,” “men have uncontrollable sexual urges,” “it is not rape because she didn’t say no,” “she falsely accused him of rape because she wants his money,” etc.) (Burt, 1980; Payne et al., 1999; Flood and Pease, 2009). Such beliefs prevent victims from reporting the assaults, and sometimes also prevent them from identifying that the assault was an actual sexual aggression (Weiss, 2009). Indeed, feelings of guilt, denial and shame, as well as the legitimate fear of being blamed and stigmatized, are still extremely prevalent among victims, who find it very challenging to talk about the assault (Ahrens, 2006). Even professionals such as clinicians or law officers are far from being immune to such prejudices and do not offer the safe space that would help victims recover thanks to adapted care and legal action (Oram, 2019).

The last decade, however, has seen a steady change taking place: there is a growing movement of victims who have found strength in numbers through social media. They have become able to face social stigma in order to report what happened to them (e.g., child abuse in church, abuse on women in the entertainment industry, abuse on teenagers in competitive sports, etc.). Such social movements might be beneficial for victims in that they realize that they are not alone and can find support. They might also be beneficial for society in that they help acknowledging the extent of the problem, since the massive amount of testimonies published everywhere demonstrate that sexual abuse victims are much more numerous than previously believed.

Statistics on prevalence in the general population indicate elevated rates of victimization all over the world. The recent WHO publication might be the most reliable reference on this topic (WHO, 2021). It states that “30% of women aged 15 years and older have been subjected to physical and/or sexual violence from any current or former husband or male intimate partner, or to sexual violence from someone who is not a current or former husband or intimate partner, or to both these forms of violence at least once” (p. XVI). In a recent study, Sardinha et al. (2022) ran meta-analyses of 366 eligible studies, representing responses of 2 million women aged 15 and older from 161 countries and areas. They found that “27% (uncertainty interval [UI] 23–31%) of ever-partnered women aged 15–49 years are estimated to have experienced physical or sexual, or both, intimate partner violence in their lifetime, with 13% (10–16%) experiencing it in the past year before they were surveyed” (Sardinha et al., 2022). In addition to instances of sexual assault during adulthood, one must also consider victimization during childhood: rates of sexual violence on children reach 12.7% (18% for girls and. 7.6% for boys), according to a meta-analysis by Stoltenborgh et al. (2015).

### Methodological Considerations Regarding Assessment of Sexual Victimization

Sexual victimization rates represent about one woman out of three and one child out of ten. Readers may not have expected such elevated figures, especially since many previous studies have reported lower rates (although a few studies actually reported higher numbers) (Dworkin et al., 2017). This is because estimates of sexual violence depend on (i) the definition of sexual violence; (ii) the choice of investigation methods (analysis of formal complaints in police stations, clinical reports, telephone interviews, internet surveys, open questions, etc.); and (iii) the country where the investigation took place. Definitions of sexual violence are obviously crucial when assessing levels of victimization. For example, in their review of literature, Dworkin et al. (2017) state that “17–25% of women and 1–3% of men will be sexually assaulted in their lifetime.” In contrast, Cleere and Lynn (2013) report much higher figures in their study of 302 college women, with 60.93% of their sample having experienced some form of sexual aggression, including but not limited to unwanted sexual contact. Regarding country-related variations, rates may differ from one country to another. For example, in France, rates of victimization were higher for men (3.9%) and lower for women (14.5%) as compared to other countries. Such figures resulted from Hamel et al. (2016) the large-scale “Violence and Gender Relations” (VIRAGE) survey that was conducted by the French Institute for Demographic Studies (INED) in order to assess lifetime levels of sexual aggression (rape, attempted rape, unwanted sexual touching) in a sample of almost 16,000 women and 12,000 men, aged 20–69 years old.

Despite this geographical and methodological variability, the WHO (2021) study is actually very consistent with two other large-scale studies that were conducted in the United States of America. The National Crime Victimization Survey (NCVS) was made using two methods of identification: self-report versus interview, randomly assigned to a sample of 11,000 women aged 18–49, in order to assess rates of sexual aggression over the past 12 months. Their overall results show that 8.1% of participants had suffered sexual violence over the past year. Their analyses show that self-report led to slightly higher rates of reported assaults than interviews (Cantor et al., 2021). The other large United States-based study was titled “National Intimate Partner and Sexual Violence Survey.” It was conducted on more than 12,000 persons by the National Center for Injury Prevention and Control (CDC). Phone interviews regarding lifetime experience indicate that rape affects 19.3% of women (and 1.7% of men), other forms of sexual violence affect 43.9% of women (and 23.4% of men) (Breiding et al., 2014).

### Proportion of Victims of Sexual Abuse in the Autistic Population

Several researchers have investigated whether autistic women would exhibit higher levels of victimization than women in the general population. Given that being on the autism spectrum condition is characterized by experiencing difficulties in social communication, such as decoding hidden intentions and emotions of others, understanding implicit communication and elements of context, it is expected that women on the spectrum may be at considerable risk for sexual victimization, a hypothesis confirmed by all published studies on this topic. To this end, we have attempted here to conduct an exhaustive review of such research.

#### Studies of Sexual Victimization in the Overall Autistic Population

Brown-Lavoie et al. (2014) in a study using an online survey, showed that 70% of autistic adults (*n* = 95) reported experiencing some form of sexual victimization after age 14 and into adulthood. In a study exploring self-reported experiences of many forms of victimization and perpetration, researchers found that adults on the autistic spectrum (*n* = 45) were more likely to report several forms of victimization, including sexual violence, than control participants (*n* = 42) (Weiss and Fardella, 2018). In a Danish national survey on violence and discrimination among people with different types of disabilities, 4 out of 23 autistic participants reported sexual victimization (Dammeyer and Chapman, 2018). In a study investigating behavioral effects of sexual molestation (as reported by caregivers) in *n* = 156 children on the autism spectrum, the authors found that 16.6% of their sample had been sexually molested and that those children were ten times more likely than the others to act out in a sexual way, a well-known behavioral consequence of such abuse (Mandell et al., 2005). In another study examining reports provided by caregivers about autistic children and teenagers (*n* = 350), it was found that 10% of the sample had been sexually molested (Brenner et al., 2018). Interestingly, the Brenner et al. (2018) study stands out in that it is the only one that represents the full autistic spectrum, with 42% of their sample falling below the IQ cutoff (70) for Intellectual Disability and 36% of the sample presenting with very low verbal ability. It must be noted, though, that reports by caregivers are not as reliable as personal reports. This is illustrated in a study of 100 dyads of young autistic adults and their parents: 62% of the young adults reported some level of victimization as compared to 54% of their parents (Hartmann et al., 2019).

#### Studies of Sexual Victimization in Female and Queer Autistic Population

However, those six studies cited above did not report whether there were differences between male and female participants. Yet, since women are disproportionately more at risk of sexual victimization than men in the general population, it seems very likely that a similar difference would be observed within the autism spectrum. Indeed, in a large scale Swedish longitudinal twins’ study, 18 years old autistic female participants presented an “almost three times increased risk of self-reported coercive sexual victimization.” Female participants with ADHD were also more victimized than controls, with a two times increased risk (Gotby et al., 2018). In research combining psychometrics and in-depth interviews about the inner experience of being an autistic adult woman, 9 out of 14 participants reported rape by their romantic partner (*n* = 7) and/or by strangers (*n* = 3) (Bargiela et al., 2016). In a study of college students involving nine campuses, participants on the autistic spectrum (*n* = 158) were twice as likely to report unwanted sexual contact as compared to students without ASC, but the analysis was limited by the small size of the group of victimized autistic participants (*n* = 13). Interestingly though, out of those 13 victims, 8 were female and 3 were non-binary, despite the autistic sample including more males (*n* = 93) than female (*n* = 44) and non-binary (*n* = 21) participants (Weiss, 2009). Of note, participants with ADHD were also very much victimized, as in the previously mentioned study by Gotby et al. (2018). In a large-scale study, autistic traits and PTSD symptoms were measured in 1247 adult women, who also reported whether they had been abused during childhood. The authors found that participants “in the highest versus lowest quintile of autistic traits were more likely to have been sexually abused in childhood (40.1% versus 26.7%) […] and to have been pressured into sexual contact (25.4% versus 15.6%) […]. High levels of PTSD symptoms were more prevalent in the highest versus lowest quintile of autistic traits (6–7 PTSD symptoms, 10.7% versus 4.5%)” (Roberts et al., 2015). In a large-scale study investigating how childhood trauma is related to self-harm in adulthood, there was a subgroup of *n* = 150 individuals on the autism spectrum. Analyzing that data, Warrier and Baron-Cohen (2021) found that autism is strongly correlated with sexual molestation during childhood, and that this effect was even much stronger in women. Recent research by Pecora et al. (2019, 2020) focused on two major intersecting risk factors for sexual victimization: sexual orientation and gender identity. It is well established that members of the LGBTQIA++^3^ community are still very much persecuted and attacked in most countries. Autistic individuals assigned female at birth are overwhelmingly part of the queer community, to the point that women who are *cis*-gender and strictly heterosexual actually constitute a minority on the autism spectrum (Greenberg et al., 2018; Pecora et al., 2019, 2020). Therefore, exploring “the nature of negative experiences in autistic and non-autistic females across gender identity and sexual orientation” was a much-needed investigation in order to understand the complexity of sexual and sentimental life of these persons. In their 2019 study (135 autistic females, 96 autistic males, 161 non-autistic females), they found that autistic females expressed less sexual interest than autistic males but not less than non-autistic females. The authors categorized negative sexual experience as (1) sexual experiences that were later regretted; (2) unwanted sexual experiences; and (3) being the victim of an unwanted sexual advances. While the methodological pertinence of such categorization is questionable, results are highly significant: autistic females were 7.15 and 2.52 times more likely to undergo negative sexual experience than autistic males and non-autistic females, respectively (Pecora et al., 2019). In their 2020 study, they included *n* = 134 autistic persons assigned female at birth and brought up even more important findings: their autistic sample was 70% queer; being autistic represented a 2.38 fold increased risk of negative sexual experience as compared to controls; within the spectrum, homosexual persons had a 3.29 fold increased risk as compared to heterosexual ones (Pecora et al., 2020). The last study that we reviewed was recently published by Joyal et al. (2021). They included 68 (27 females) young adults on the autism spectrum. Their research show that, regarding negative sexual events, young autistic women are four times more likely than young autistic men and twice as likely than non-autistic women to undergo such experience. Very interestingly, the researchers found out that, within the autistic spectrum, being a (a) late diagnosed (b) female with (c) sufficient education about sexuality and (d) desire for sex were at the same time strong predictors of positive sexual experience and strong predictors of negative sexual experience.

#### Elevated Risks of Sexual Victimization in Autistic Individuals Identified as Female

We tentatively conclude from this short review that being autistic means undergoing a 10–16% risk of enduring sexual molestation as a child and a 62–70% risk of being sexually victimized in adulthood. Most victims are girls and women: autistic female risk of being sexually assaulted is between two and three times as much than non-autistic females and about four times as much than autistic males. Those figures are consistent with the general population rates: around 30% of women and 12% of children are sexually victimized in their lifetime.

In the line of this research, we have conducted an internet-based survey in order to measure the prevalence of sexual victimization in women on the autism spectrum (*n* = 225) by using standardized assessment. Thereby, we have recruited participants to our survey through the websites of non-profit organizations in order to investigate their lifetime prevalence exposure to sexual aggression and abuse, their autistic traits, as well as comorbid psychiatric disorders for which they were treated (depression, anxiety disorders) and behaviors that can be connected with childhood or adult sexual abuse (i.e., suicide attempts, self-mutilations, substance abuse…) insofar as these behaviors may represent “red flags” that can alert relatives and clinicians. Our study first aim was to confirm previous results using a larger sample. More importantly, our goals were to expand such results by investigating several dimensions of sexual victimization that bear clinical and social significance, such as age at first assault, that we hypothesized to be correlated to levels of revictimization. We also used two modes of inquiry (directed versus open question) because we expected that they would yield different results. Last, we explored other dimensions such as health consequences of aggression, with a focus on post-traumatic stress-disorder, and follow-ups of the aggression such as reports and opinions of victims regarding prevention methods. Importantly, in our study, sexual victimization was defined as one or several of the three following situations: unwanted sexual contact; rape; attempted rape. Our definition was therefore limited and did not include other forms of sexual violence such as sexual harassment, stalking, indecent exposure, revenge porn, etc.

## Materials and Methods

This internet-based study was conducted in France in April and May 2018. A total of 234 participants completed the questionnaire. Our final sample was *n* = 225, since 9 participants did not meet inclusion criteria (3 did not provide consent for their data to be used; 2 obtained a Ritvo Autism and Asperger Diagnostic Scale (RAADS) score less than 14; 4 were aged less than 18).

In order to facilitate results replication and refutation, an extended and detailed version of the statistics section is provided as Supplementary Material, as well as an English translation of the questionnaire.

Repository URL for R code is: https://nakala.fr/10.34847/nkl.4021h29a.

Repository URL for dataset is: https://nakala.fr/10.34847/nkl.d3d35i2o.

### Recruitment of Participants

Due to social and communication difficulties, many women on the autism spectrum join online autism communities where they can find help and support from peers and where they may feel more comfortable discussing issues of sexual violence that they have experienced. We have contacted several local organizations dedicated to autism (*Association Francophone des Femmes Autistes, Asperger Amitié, Asperger Aide, France Asperger*…) and asked them for their support by sharing to their community an internet link to a data collection site.

### Procedure

After a short description of the study, women were asked to participate in this survey if they were identifying as autistic women. Participants provided consent for their data to be used in the study after completing anonymously the online questionnaire. A trigger warning was included before questions regarding victimization, as well as an invitation for victims to contact a health practitioner. After pre-submission, the president of the ethics committee of Paris V decided that it was not necessary to review the study insofar as the participants were strictly anonymous (no identifying data were collected, nor IP addresses of respondents). As per French laws regarding data privacy, the questionnaire was declared to CNIL authorities (National Commission on Informatics and Liberty) under identifier # 2172655.

### Measures

All participants answered to three questionnaires, for a total of 38 questions, using an online survey system^4^. All questions were multiple choice questions with the exception of questions #8, #13, #33, #36, #37 which were multiple response questions (i.e., “check all that apply”). In addition, questions #8, #9, #13, #33, #34, #36, #37 included an open field question: “Other (please specify).”

#### Scope of Questions

∘ Questions #1–6: socio-demographic information.
∘ Questions #7–9: health level and A.S.D diagnosis.
∘ Questions #10–12: sexual activity and sexual orientation.
∘ Question #13: victimization status in an open question form allowing multiple responses. Choices were as follows: (1) I haven’t been sexually assaulted; (2) I have been sexually assaulted; (3) I have been raped; (4) I underwent an attempted rape; (5) I do not know; (6) Other (with open field).
∘ Questions #14–27: autistic traits (RAADS-14 scale) (Ritvo et al., 2011).
∘ Questions #28–31: victimization level (items 1, 2, 3, and 5 of the Sexual Experiences Survey (SES) scale – Short Form Victimization) (Johnson et al., 2017).
∘ Questions #32–33: age at first assault and revictimization status.
∘ Question #34: for victims, did they report the assault (with no specification to whom)? And what were the consequences of reporting (being believed or not; receiving psychological care or not; filing a complaint or not)?
∘ Question #35: for victims, what could have prevented the assault? (listed options were chosen by the authors based on clinical interviews).
∘ Question #36–37: presence of psychiatric disorders and assessment of consequences following the assault.
∘ Question #38: participants’ consent to have their responses used in this study.

#### Measure of Autistic Traits With RAADS-14 Scale

The RAADS scale is considered to be one of the most reliable self-report screening tools for ASD. In the validation study of the RAADS-14 based on the 80 item Ritvo Autism and Asperger Diagnostic Scale-Revised [RAADS-R (Ritvo et al., 2011)], the median score for ASD was 32, and 11 for other psychiatric disorders, whereas a cut-off score of 14 or above reached a sensitivity of 97% and a specificity of 46–64%.

We calculated the mean, median and standard deviation of the RAADS scores across our sample of participants. We also calculated scores for the three sub-components of RAADS: “social communication” (columns 1, 4–6, 8, 9, and 11); “hyper-focalization” (columns 12–14) and “sensory reactivity” (columns 7 and 10).

#### Measure of Sexual Victimization With Sexual Experiences Survey Questionnaire

In order to assess victimization status of participants, we used a French translation of items 1, 2, 3, and 5 of the Sexual Experiences Survey Short Form Victimization questionnaire (SES-SFV). The Sexual Experiences Survey (SES) and its recent 10-items update (SES-SFV) are the most widely used measures of unwanted sexual experience (Johnson et al., 2017). The fact that it consists of questions about specific situations is important, since responders may not realize that their experiences could be classified as victimization.

Of important note, we did not apply the recommended analysis method for answers to SES-SFV, which distributes them into six situations: non-victim, sexual contact, attempted coercion, coercion, attempted rape and rape. This is due to the fact that the distinction between coercion and assault does not exist in France, where sexual relationships obtained through coercion are legally categorized as rape in France. Therefore, we have used the following categories instead: sexual touching (s1), oral rape (s2), vaginal/anal rape (s3) and attempted rape (s4). In our analyses, (s2) and (s3) were pooled together in a single category titled “rape.”

### Statistics

Statistical analyses were realized with R (R 4.1.1 2021.08.10). We used the following methods^5^ : descriptive statistics (percentages, means, medians and standard deviations). We used non-parametric statistical analyses because data was mostly non-continuous. We used Chi square test to assess for significant differences between subgroups. When applicable, we also used McNemar test and conditional probabilities in order to confirm Chi square tests’ results, because differences in sample sizes of our subgroups could increase type 1 and type 2 errors. In order to assess whether some characteristics of our sample increased measured risks (e.g., developing Post Traumatic Stress Disorder), we used three methods: two-sample test for equality of proportions, Wilcoxon test and univariate logistic regression with odds ratio and confidence intervals. Unless otherwise specified, we used Yates continuity correction as *post hoc* correction in all cases. Bonferroni correction was additionally used when applicable.

## Results

### Population Description

#### Socio-Demographics

All participants declared themselves female except one. Participants’ characteristics are reported in Table 1.

**TABLE 1:** Socio-demographic characteristics of participants.

Socio-demographic characteristics	*N* (%)
**Age range**	
18–20	4 (1.8%)
21–29	47 (20.9%)
30–39	91 (40.4%)
40–49	62 (27.6%)
50–59	17 (7.6%)
60 and more	4 (1.8%)
**Education levels**	
No diploma	5 (2.2%)
Grade school	1 (0.4%)
Junior high school	7 (3.1%)
High school (no diploma)	22 (9.8%)
High school diploma	43 (19.1%)
University – License degree (3 years)	67 (29.7%)
University – Master’s degree (5 years)	67 (29.7%)
University – Doctoral degree (8 years)	13 (5.7%)
**Occupation**	
Student	23 (10.2%)
Employee	41 (18.2)
Executive	25 (11.1%)
Independent worker	25 (11.1%)
Unemployed – looking for a job	23 (10.2%)
Unemployed – not looking for a job	17 (7.6%)
Disability payments	23 (10.2%)
Sick leave or inability to work	31 (13.7%)
Other (please specify)	17 (7.6%)

#### Diagnosis and Comorbidities

In our sample, 60 (26.7%) participants were self-diagnosed, 143 participants (63.6%) declared having received professional diagnosis, and the remainder 22 (9.8%) participants declared situations ranging from early questioning to being currently in the diagnosis process. Hundred and forty-nine participants (66.2%) received IQ testing: 25 (16.7% of those whose IQ was assessed) reported average IQ scores [90–119], 45 (30.2%) reported above average scores [120–129] and 78 (52.3%) reported IQ > 130.

Participants reported their state of health as good to excellent for 119 (52.9%) of them, while 67 (29.8%) rated their health as average and 39 (17.3) reported bad health state. Participants also reported existing comorbidities as follows:

• Depression: 140 (62.2%)
• Anxiety: 127 (56.4%)
• Post-traumatic Stress Disorder (PTSD): 60 (26.7%)
• ADHD: 25 (11.1%)
• Bipolar disorder: 15 (6.7%)
• Borderline personality disorder: 12 (5.3%)
• Substance abuse: 9 (4.0%)
• Alcohol abuse: 8 (3.6%)
• Schizophrenia: 2 (<1%)

Regarding comorbidities, participants could declare more than one existing comorbidity as shown in Table 2.

**TABLE 2:** Number of comorbidities.

Number of comorbidities	0	1	2	3	4	5
Number of participants	56 (24.9%)	35 (15.6%)	59 (26.2%)	59 (26.2%)	12 (5.33%)	4 (1.8%)

#### Sex Life

Table 3 provides description of participants’ sex lives. Out of 125 individuals who declared having been sexually active over the last six months, 96 (77%) were in a relationship/married, while 29 (23%) were not in a relationship/married.

**TABLE 3:** Sex life characteristics of participants.

Sex life characteristics	*N* (%)
**Relationship status**	
Single	68 (30.2%)
In a relationship	80 (35.6%)
Married	50 (22.2%)
Divorced/separated	26 (11.6%)
Widow	1 (0.4%)
**Sexual orientation (sexually attracted by)**	
Opposite sex only	64 (28.4%)
Bisexual	135 (60.1%)
Same sex only	3 (1.3%)
Asexual	23 (10.2%)
**Sexual experience with (over lifetime)**	
Opposite sex only	122 (54.2%)
Bisexual	86 (37,5%)
Same sex only	5 (2.2%)
Asexual	12 (5.3%)
**Frequency of sexual relationships over the last six months**	
I have not had any sex	100 (44.4%)
I have sex less than once a month on average	33 (14.7%)
I have sex between once and three times a month on average	41 (18.2%)
I have sex between four and ten times a month on average	36 (16.0%)
I have sex more than ten times a month on average	15 (6.7%)

### Measure of Autistic Traits (Ritvo Autism and Asperger Diagnostic Scale)

#### Description of Ritvo Autism and Asperger Diagnostic Scale Results

RAADS total scores: mean = 34.88; median = 36.00; *SD*: 5.24.

RAADS sub scores social communication: mean = 16.06; median = 17.00; *SD* = 3.61.

RAADS sub scores hyper focalization: mean = 8.05, 9, 1.64; median = 9.00; *SD* = 1.64.

RAADS sub scores sensory reactivity: mean = 5.30; median = 6.00; *SD* = 1.30.

**FIGURE 1:**
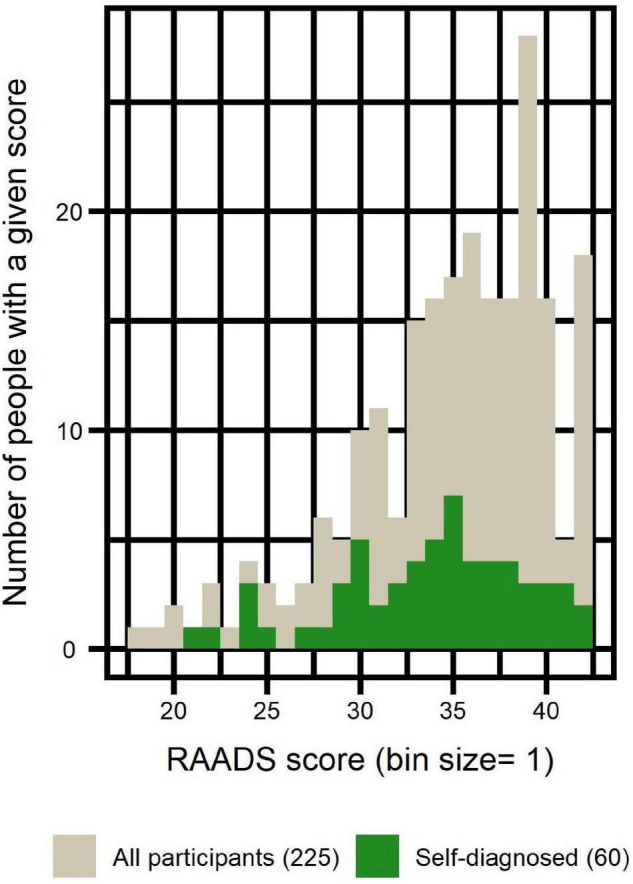
Distribution of RAADS scores of self-diagnosed participants compared to the distribution of all participants.

Figure 1 illustrates the comparison of self-diagnosed participants to the whole sample in terms of RAADS scores distribution.

### Sexual Victimization

#### Description of Q13 Results: Open Question About Victimization Status

Hundred and fifty-five (68.9%) participants declared being victim of one or another form of sexual violence. 22 (9.8%) participants declared not having been victimized while 39 (17.3%) declared that they did not know. Declared victims were distributed as follows: assault: 117 (52.0%); rape: 69 (30.7%); attempted rape: 23 (10.2%).

#### Description of Sexual Experiences Survey Results: Guided Questions About Victimization Status

Hundred and ninety-nine (88.4%) participants reported being victim of one or another form of sexual violence. Twenty-six (11.6%) participants declared not having been victimized. Reported victims were distributed as follows: sexual touching and/or rape attempt: 196 (87.1%); rape: 155 (68.9%).

**FIGURE 2:**
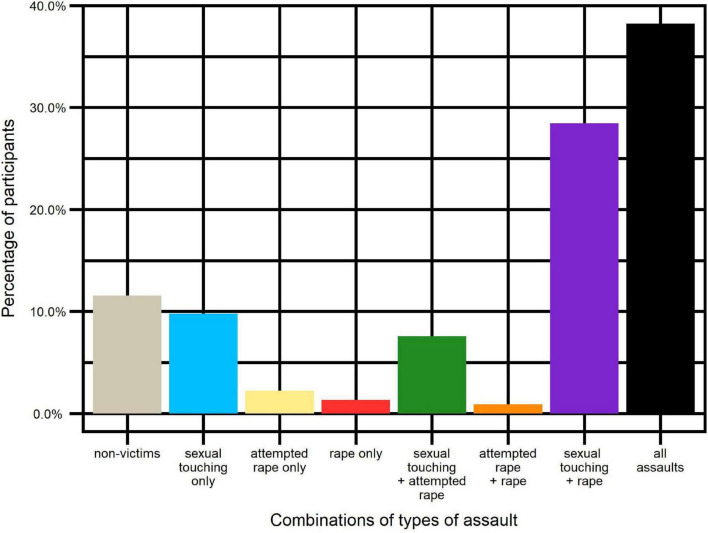
Percentage of participants in combinations of types of assaults.

While some participants reported only one type of aggression, others reported being victims of several types of aggressions, as illustrated in Figure 2:

Victims of sexual touching only: *n* = 22 (9.8%).

Victims of attempted rape only: *n* = 5 (2.2%).

Victims of rape only: *n* = 3 (1.3%).

Victims of several categories of assaults: *n* = 169 (75.1%).

Non-victims: *n* = 26 (11.6%).

#### Description of Q32–33: Age at First Aggression, Frequency of Abuses and Cases of Multiple Offenders

Age at first aggression was distributed as follows:

• Less than 9 years old: 43 (19.1%)
• Between age of 10 and 12: 36 (16.0%)
• Between age of 13 and 15: 33 (14.7%)
• Between age of 16 and 18: 23 (10.2%)
• Between age of 19 and 30: 59 (26.2%)
• More than 30 years old: 5 (2.2%)
• Non-victims: 26 (11.6%)

Cases of multiple aggressions were distributed as follows (choices could be cumulative):

• There was only one case of aggression: 30 (13.3%)
• It happened several times with the same offender: 91 (40.4%)
• It happened several times with several offenders: 126 (56.0%)
• Non-victims: 26 (11.6%)

#### Description of Strategies Used by Perpetrators, With Detailed Description Depending on the Type of Aggression

**FIGURE 3:**
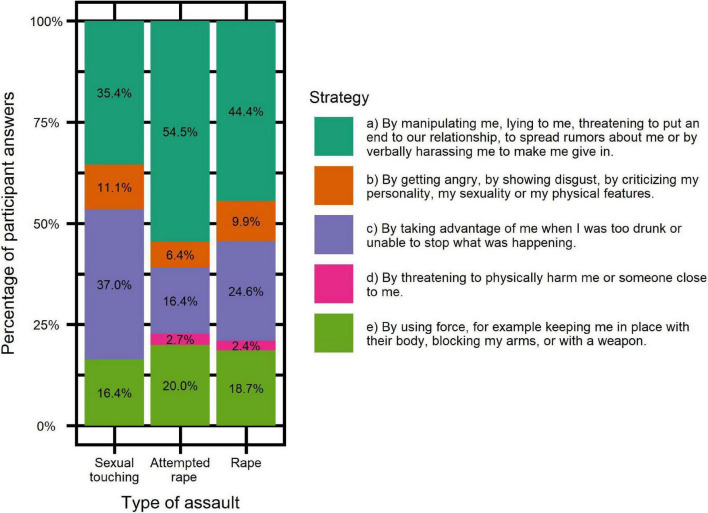
Strategies used depending on the type of assault.

Participants reported that offenders used different strategies depending on the type of aggression, as illustrated in Figure 3. The Cronbach’s alpha was 0.7 and the McDonald’s omega total was 0.74.

#### Description of Q37: Consequence of Aggression at 6 Months

**FIGURE 4:**
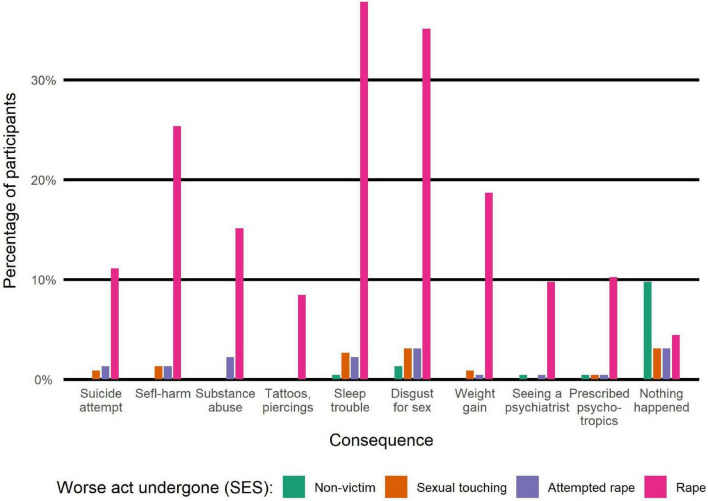
Consequences of assault within 6 months of happening.

Participants reported consequences of the aggression during the 6-month period following the assault. As illustrated in Figure 4, distribution of such consequences among victims (according to SES questionnaire) is as follows:

• Sleep disorder: 96 (48.2%)
• Disgust for sex: 93 (46.7%)
• Self-harm: 63 (31.7%)
• Weight gain: 45 (22.6%)
• Drugs and/or alcohol abuse: 39 (19.6%)
• Suicide attempt: 30 (15.1%)
• Tattoo/piercing: 19 (9.5%)

#### Description of Q34–35: Help Requests and Prevention Opinions

Among the 199 victims, 111 (55.8%) did not report the assault. Among the 69 who told others about the assault, 18 (26.1%) were not believed. Among the 51 who reported the assault and who were believed (25.6% of the victim total), 34 (66.7%) received no care and no complaint was filed, 8 (15.7%) received care and a complaint was filed, 4 (7.8%) did not receive care but a complaint was filed, 5 (9.8%) received care but no complaint was filed. The remainder of victims either answered “other” (16, 8%) or were among the 29 participants (12.9% of the full sample) who answered “not applicable” alongside the non-victims.

Participants also chose which of the following sentences best reflected their own opinions regarding prevention methods:

• Sexual manipulators or predators seem to spot you more easily because of your difficulties in sexual interactions: *n* = 50 (22.2%).
• Knowledge of self-affirmation strategies learned in therapy (or social skills groups) could have helped you stay safe better: *n* = 39 (17.3%).
• Your family members should have been more attentive: *n* = 37 (16.4%).
• Female teenagers with Asperger syndrome and their parents should be better informed about the risk of sexual abuse: *n* = 23 (10.2%).
• Nothing could have protected you from this or these assault(s): *n* = 24 (10.7%).
• Health professionals should have given you adapted prevention advice such as better identifying sexually ambiguous situations with men: *n* = 18 (8.0%).
• The officials of the institution (high school, university, company) in which you were should have been more watchful: *n* = 8 (3.6%).
• Not applicable (this is not relevant to me): *n* = 26 (11.6%).

#### Hypothesis #1: Open vs. Guided Questions (Question#13 vs. Sexual Experiences Survey Questionnaire) Yield Different Results

In order to compare answers from the two sexual assault questionnaires, we checked overlaps and incongruencies between Question#13 and SES questionnaire. We looked at how many participants who had chosen at least one of the positive answer columns (the assault, rape and attempted rape options) in the open question were also victims according to SES, checking the same for participants who hadn’t. We also looked at how many participants who didn’t know or declared being non-victims in the open question were nevertheless victims according to SES, and how many weren’t.

While 153 (68%) participants reported victimization in both SES and question#13, there were 46 (20.4%) participants who did not identify as victims in question#13 but nevertheless were classified as victims according to the SES questionnaire. On the other hand, only 2 (<1%) participants reported being victims when answering to question#13 but were not categorized as victims according to SES questionnaire.

Also, among the 39 participants who answered “I don’t know” to question#13, 34 of them were actually categorized as victims according to SES questionnaire. More precisely, those 34 victims reported one or more of the following assaults: Sexual touching: 31 (91.2%); rape: 22 (64.7%); rape attempt: 16 (47.1%).

**FIGURE 5:**
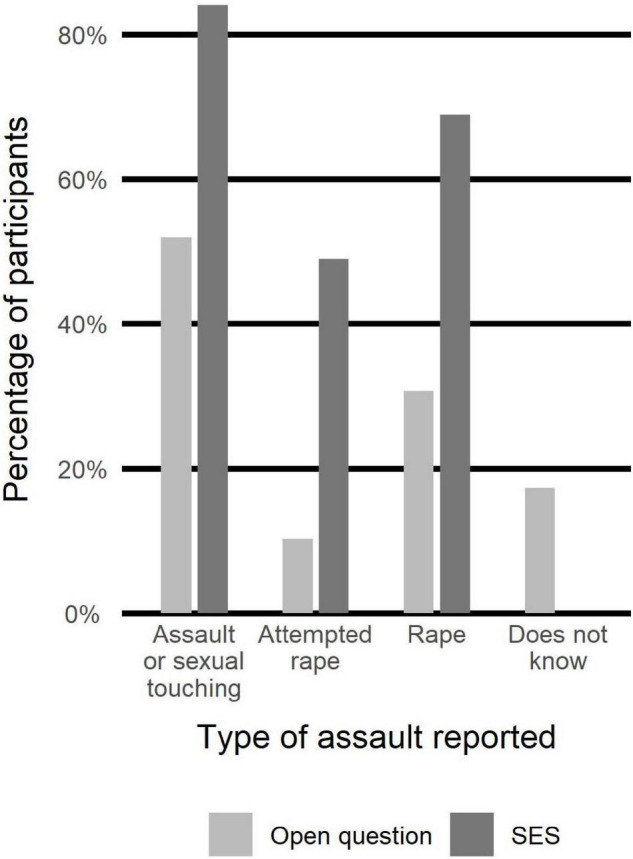
Comparison of assaults reported by participants through the two questionnaires.

Moreover, among the 22 participants who answered being non-victims to question#13, 5 were recategorized as victims according to SES questionnaire, distributed as follows: Sexual touching: 3 (60%); rape 1 (10%); rape attempt: 4 (80%). Those results are illustrated in Figure 5.

In order to check consistencies between the two modes of investigation (question#13 and SES), we used two methods: Chi-square tests and Mc Nemar tests were run on the contingency tables.

Chi squared tests results were as follows and remain significant after a Bonferroni correction:

- Sexual touching (SES) or assault (open question): *p* = 2.27e-07*
- Rape: *p* = 2.01e-08*
- Rape attempt: *p* = 4.61e-05*

McNemar tests results were as follows:

- Sexual touching (SES) or assault (open question): *p* = 2.05e-15
- Rape: *p* < 2.2e-16
- Rape attempt: *p* < 2.2e-16

#### Hypothesis #2: Being Victim of One Aggression Increases the Risk of Further Aggressions

In order to investigate whether being victim of one assault increases the risk of revictimization, we used conditional probabilities within SES questionnaire, with the following results:

- *P*(sexual touching | rape) = 96.8%
- *P*(sexual touching | attempted rape) = 93.6%
- *P*(rape| sexual touching) = 79.4%
- *P*(rape| attempted rape) = 80.0%
- *P*(attempted rape| sexual touching) = 54.5%
- *P*(attempted rape| rape) = 56.8%

We ran Chi-square tests on the contingency tables within SES questionnaire (see Table 4), with the following results, which remain significant after a Bonferroni correction:

**TABLE 4:** Contingency tables for types of assault two by two.

		Rape		Attempted rape	
		Non-victim	Victim	Non-victim	Victim
Sexual touching	Non-victim	31 (13.8%)	5 (2.2%)	29 (12.9%)	7 (3.1%)
	Victim	39 (17.3%)	150 (66.7%)	86 (38.2%)	103 (45.8%)
Attempted rape	Non-victim	48 (21.3%)	67 (29.8%)		
	Victim	22 (9.8%)	88 (39.1%)

- Sexual touching and rape: *p* = 2.27e-07*
- Sexual touching and rape attempt: *p* = 0.0002*
- Rape and rape attempt: *p* = 0.0007*
- Sexual touching and rape and rape attempt: *p* = 1.72e-14*

We also ran McNemar tests on the contingency tables within SES questionnaire, with the following results:

- Sexual touching and rape: *p* = 6.53e-07*
- Sexual touching and rape attempt: *p* = 6.05e-16*
- Rape and rape attempt: *p* = 3.10e-06*

#### Hypothesis #3: Being Young at First Aggression Increases the Risk of Further Aggressions

**FIGURE 6:**
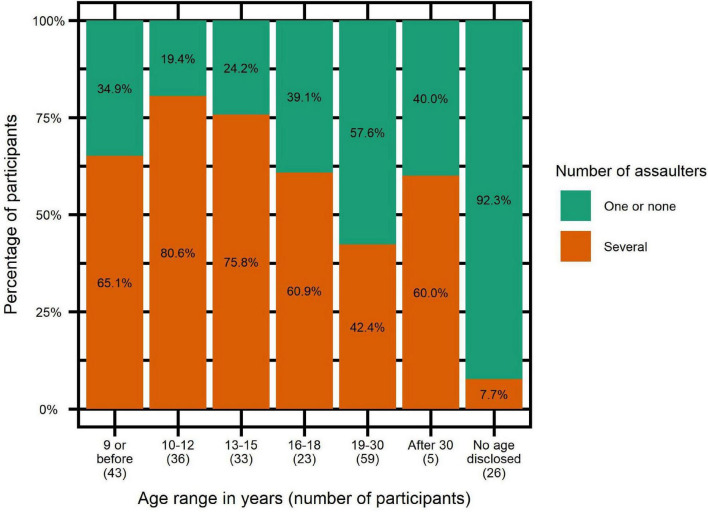
Percentages of participants with several abusers depending on age at first assault.

We investigated whether the age at first assault is related to the risk of revictimization by another offender, as shown in Figure 6.

We did a logistic regression in which having had several abusers was the dependent variable and the age of first assault the independent variable, with the following results: *P*-value = 0.0027*; Odds-ratio = 0.75; confidence interval = (0.62–0.90). The influence of age of first assault remained when controlling for PTSD and presence of several assaults by the same offender, with the latter also showing significant correlation with the dependent variable.

We ran another logistic regression in which being victim of several assaults by the same offender was the dependent variable and the age of first assault the independent variable, with the following results: *P*-value = 0.0513; Odds-ratio = 0.84; confidence interval = (0.70-1.00). The influence of age of first assault increased to the point of becoming significant when controlling for PTSD and presence of several offenders, with the latter also showing significant correlation with the dependent variable.

#### Hypothesis #4: Perpetrators Use Different Aggression Strategies Depending on Aggression Types

We investigated whether offenders used different aggression strategies depending on the type of assault. Chi-square tests were done on a contingency table within SES of all five strategies and all types of assault (see Tables 5, 6) to see if there were any differences in the distribution of strategies between the three types of assault. Importantly, since strategy “*d* = threatening physical harm” was so rare, we had to exclude it from the contingency tables in which it would have been its own category, but kept it when it was a component of a larger category. Of note, we also ran those tests without Yates correction, with comparable results. As a reminder, the remaining strategies are: manipulation/mental ascendency/harassment (“a”), anger/disgust/malicious criticism (“b”), using surprise to take advantage (“c”) and use of force (“e”). The *p*-values that are significant remain so after a Bonferroni correction.

**TABLE 5:** Numbers used in the single strategy centered contingency tables.

	Sexual touching victim answers		Attempted rape victim answers		Rape victim answers	
	Used	Not used	Used	Not used	Used	Not used
Strategy a	67	122	60	50	112	140
Strategy b	21	168	7	103	25	227
Strategy c	70	119	18	92	62	190
Strategy e	31	158	22	88	47	205

**TABLE 6:** *p*-Values of difference in presence of strategies depending on aggression type.

	All three types of aggressions	Sexual touching and rape attempt	Rape and rape attempt	Sexual touching and rape
Strategy a	0.0051*	0.0019*	0.0978	0.0710
Strategy b	0.3963	0.2489	0.3707	0.8046
Strategy c	0.0002*	0.0003*	0.1096	0.0066*
Strategy e	0.7101	0.5296	0.8767	0.6267

*p < 0.05.

#### Hypothesis #5: History of Sexual Aggression and the Risk of Post-traumatic Stress Disorder Are Correlated

Participants could declare one or more existing comorbidities. The mean number of declared comorbidities was different depending on victimization. We also report here the details of comorbidities declared by each group (see Table 7). In order to test if victimization increased the number of comorbidities, we used Wilcoxon test to compare mean number of comorbidities in victims versus non-victims, resulting in *p* = 0.016*.

**TABLE 7:** Consequences within 6 months of assault.

	Victims (*n* = 199, according to SES)	Non-victims (*n* = 26, according to SES)
**Number of comorbidities**		
Mean (±SD)	1.84 (±1.34)*	1.19 (±1.02)*
Median	2	1
**Types of comorbidities**		
Depression	125 (62.8%)	15 (57.7%)
Anxiety	116 (58.3%)	11 (42.3%)
Post-traumatic stress disorder (PTSD)	57 (28.6%)*^9^	3 (11.5%)*
ADHD	23 (11.6%)	2 (7.7%)
Bipolar disorder	15 (7.5%)	0 (0.0%)
Borderline personality disorder	12 (6.0%)	0 (0.0%)
Substance abuse	9 (4.5%)	0 (0.0%)
Alcohol abuse	8 (4.0%)	0 (0.0%)
Schizophrenia	2 (1%)	0 (0.0%)

^9^Of note, the difference appears to be significant only when Yates correction is not applied. *p < 0.05.

**FIGURE 7:**
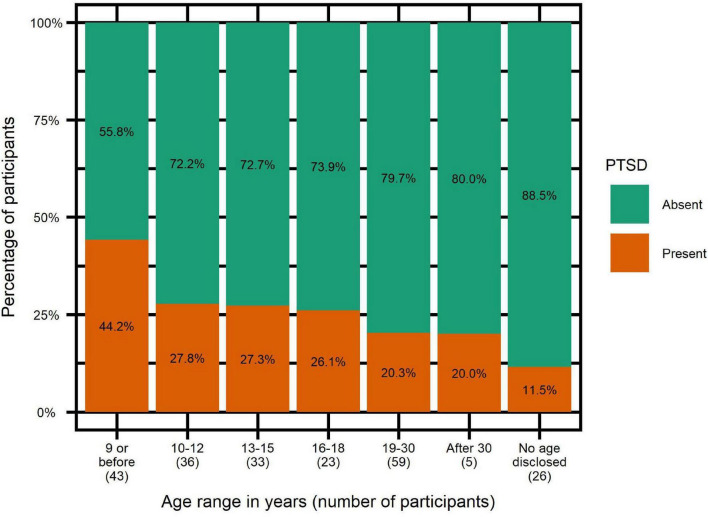
Post-traumatic stress disorder rates depending on age at first assault.

In order to test if victimization increased the risk of a specific type of comorbidity, we ran two-sample tests of equal proportions within SES, leading to the following p-values: *p* = 0.7707 for depression, *p* = 0.1817 for anxiety and *p* = 0.1054 for PTSD. Of note, the same analysis ran without Yates correction led to significant p-value for PTSD only: *p* = 0.0467*. Figure 7 illustrates the percentage of PTSD in victims depending on age at first assault.

In order to test whether SES victimization increased the risk of PTSD, we ran logistic regressions with the following results: *p*-value = 0.0760, odds-ratio = 3.08, confidence interval [1.02–13.33]. Using RAADS score, current age and education level as control variables revealed RAADS score to have the strongest correlation. Using the RAADS score as the sole predictor yielded the following results: *p*-value = 0.0125*, odds-ratio = 1.09, confidence interval [1.02–1.16]. Last, among SES victims, we explored whether age at first aggression was a predictor of PTSD, with the following results: *p*-value = 0.0162*, odds-ratio = 0.78, confidence interval [0.64–0.95]. Controlling for presence of several assaults from the same assaulter and presence of several assaulters, two other variables correlated with age at first assault, did not significantly change this result.

In order to investigate whether multiple assaults perpetrated by one or several offenders made a difference regarding the risk of developing a PTSD, we ran two-sample tests of equal proportions within SES, with the following results:

- Victims of several assaults from several offenders and victims of only one offender (be it one or several assaults): *p* = 0.6465.
- Victims of several assaults from same offender and other victims (several offenders + only one assault): *p* = 1.

We also ran logistic regressions among victims in order to test if the risk of developing PTSD was increased by multiple victimization by

- the same offender: *p*-value = 0.948; odds-ratio = 0.98; confidence interval [0.52–1.82]
- several offenders: *p*-value = 0.535; odds-ratio = 1.23; confidence interval [0.65–2.38]

#### Hypothesis #6: Presence of Autistic Traits Increases the Risk of Sexual Aggression

**FIGURE 8:**
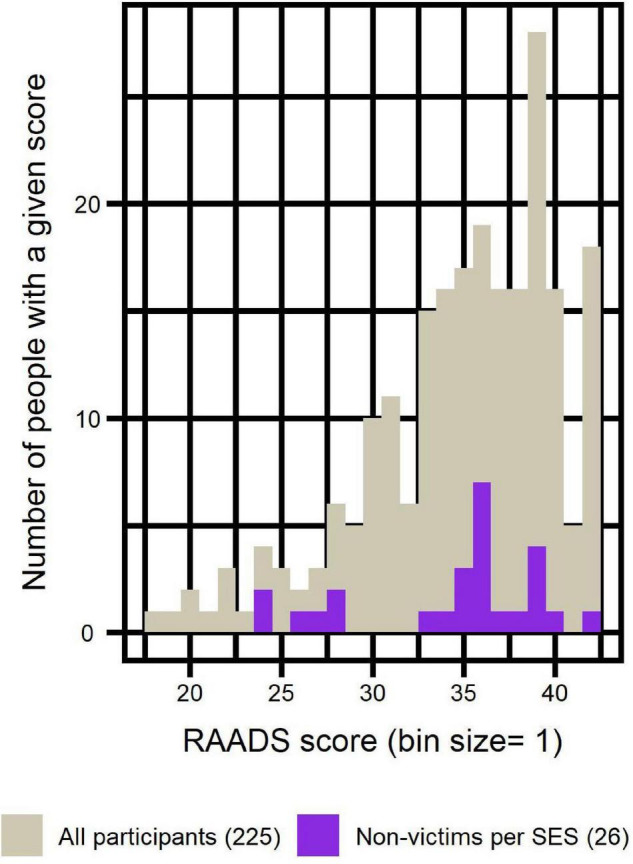
Distribution of RAADS scores of non-victims (per SES) compared to distribution of RAADS scores of all participants.

We investigated whether presentation of autistic traits constituted a risk factor for sexual victimization. In order to compare RAADS scores of victims and non-victims according to SES, we used Wilcoxon tests and logistic regressions. Adding current age and education level as independent variables to the logistic regressions did not significantly change the results. Results are illustrated in Figure 8 and reported in Table 8.

**TABLE 8:** Ritvo Autism and Asperger Diagnostic Scale score as a risk for victimization.

	Victims (*n* = 199, according to SES)	Non-victims (*n* = 26, according to SES)	Wilxocon tests	Logistic regression
RAADS total	Mean: 34.94 (±5.27)	Mean: 34.38 (±5.05)	*p* = 0.5707	*p* = 0.6080, odds-ratio = 1.02, confidence interval [0.94–1.10]
RAADS “social communication” sub-scores	Mean: 15.98 (±3.67)	Mean: 16.65 (±3.14)	*p* = 0.4755	*p* = 0.3750, odds-ratio = 0.95, confidence interval [0.83–1.06]
RAADS “hyper focalization” sub-scores	Mean: 8.12 (±1.60)	Mean: 7.54 (±1.92)	*p* = 0.1272	*p* = 0.0986, odds-ratio = 1.19, confidence interval [0.95–1.47]
RAADS “sensory reactivity” sub-scores	Mean: 5.37 (±1.19)	Mean: 4.77 (±1.90)	*p* = 0.1354	*p* = 0.0327*, odds-ratio = 1.31, confidence interval [1.01–1.68]

*p < 0.05.

## Discussion

### Sample Description Is Consistent With Literature and Clinical Observations

The description of our population sample is revealing of what autism in women looks like and is in line with previous studies (Lai et al., 2017; Ormond et al., 2018). Participants included in our study (*n* = 225) were of all ages but predominantly in their thirties. A quarter of participants were self-diagnosed.

We asked participants if they had received IQ testing. Among the 66.2% who answered yes, 82.5%, (*n* = 123, i.e., more than half of our total sample) obtained a score > 120 and 52.3% (*n* = 78, i.e., more than a third of our total sample) reported IQ > 130. Of course, we can’t extrapolate such numbers to the whole sample, because of a potential clinical bias: it cannot be excluded that IQ testing was more often realized in individuals displaying signs of high IQ, since clinicians may have found that such measures would prove helpful to this subpopulation. Nevertheless, 34.6% of our total sample scored over 130. If true (we had no direct access to the IQ tests; recall memory bias and social desirability bias cannot be excluded), these results seem to be a rather exceptional situation, and it confirms field observations that report sharp mindedness in the population of ‘‘high-functioning’’^6^ autistic women. Two thirds of these women graduated university, but only half of them were professionally active (work or studies). Despite demonstrably high cognitive abilities and education levels, participants in our study experienced employment difficulties. This discrepancy illustrates the well-documented employment issues endured by autistic persons.

More than half of the participants (57.8%) were involved in a relationship and more than half (55.6%) had been sexually active over the last six months (mostly the same individuals, 77% of them being in a relationship while the remainder was not). Of important note, more than half of our sample had only experienced heterosexual sex over their lifetime, despite the extremely high percentage of declared queer orientations (71.6%). This gap between aspiration and experience might be partly explained by social factors that are non-specific to autistic women (i.e., cultural stereotypes, lower proportion of potential partners…), but could also be linked to autistic characteristics, such as camouflaging and social imitation strategies that have been previously described in autistic women (Lai et al., 2017). Another partial and cumulative explanation could be that since a large majority (60.1%) of our participants were actually bisexual, we may postulate that they simply settled with the most available type of romantic relationship. Of note, our results are very consistent with literature describing that sexual orientation in autistic women is very diverse (Greenberg et al., 2018; Pecora et al., 2019, 2020).

Interrogated about their health, while only 17% of participants reported poor health status, more than 75% reported at least one psychiatric comorbidity, as if mental health disorders comorbidities were not health issues. Depression and anxiety were by far the most prevalent comorbidities (62.2 and 56.4% respectively). PTSD came in third place (26.7%). These results should however be interpreted cautiously because of the absence of structured clinical interviews for psychiatric diagnosis in this study. Considering that all these findings are in agreement with reports and studies on the topic of autism in adult women, it is reasonable to see our sample as reliably representative (Lai and Baron-Cohen, 2015).

In order to confirm autism diagnosis reported by participants, we collected their RAADS scores. We only included in the analyses participants who obtained RAADS score > 14 (merely two participants were excluded solely on that criterion). Mean RAADS in our sample was equal to 34.9 ± 5.2 and median was equal to 36, well above the median = 32 reported in the validation study of that scale (Ritvo et al., 2011). Moreover, the distribution of RAADS scores of self-diagnosed participants reflected that of the clinically diagnosed, confirming that including this sub-group in the analyses was methodologically acceptable.

### Sexual Victimization Is Very High, and Victims Are Young

We assessed victimization status twice: through an open question (Question#13) and through a specific questionnaire (SES-SVF). Both methods yielded extremely high figures: open question resulted in 68.9% victimization, compared to 88.4% with SES-SFV questions. Those results are very high but nonetheless extremely consistent with the findings that we have reviewed earlier: a rate of almost 30% victimization in non-autistic women and a 2 to 3-fold increased victimization of autistic women (Roberts et al., 2015; Bargiela et al., 2016; Weiss and Fardella, 2018; Pecora et al., 2019, 2020; Joyal et al., 2021; Warrier and Baron-Cohen, 2021). Moreover, results indicate that sexual aggressions started while most of the victims were underage or barely able to legally consent: out of 199 victims, more than two thirds (68%, *n* = 135 victims) where aged 18 or below when the first aggression took place; more than half (56%, *n* = 112) being at or below age of consent (15 in France).

Regarding ascertainment procedure, we used two methods of case identification. As expected, the SES-SVF questions revealed a significantly higher level of victimization than the open question. This confirms that it is necessary to ask questions about specific situations, rather than using open questions, in order to identify all victims, since some victims may need many years of processing memories about the assault before realizing that the non-consensual sex that happened to them qualifies as a legal offense/crime. One participant wrote this “It happened many times over my lifetime (as child, as a teen, as an adult). It is only 5 years ago, at the age of 50, that I understood that I had been victim of sexual violence.” Another participant wrote “This event remained out of my consciousness’ access for many years.” We indeed found several instances of such confusion in our sample: 34 out of 39 participants who answered “I don’t know” to the open question were nevertheless identified as victims through SES. Furthermore, 5 out 22 participants who responded “non-victim” to the open question were actually victims according to SES. Last, Chi-squared test results indicated that participants were consistent: all but two of those who declared being victim in the open question declared the same thing in SES-SFV questions.

### Sexual Revictimization Is Also Very High

Sexual revictimization among our participants was very important: 84.9% of victims were revictimized (*n* = 169 out of 199). In other words, 75.1% of all participants reported several aggressions. By contrast, only 13.3% (*n* = 30) were assaulted just once over their lifetime – with the assault consisting of unwanted sexual touching for most of them (*n* = 22).

We used four different statistical methods to assess whether being victim of one assault increased the risk of being victim of another one, and all yielded significant results, reaching a clear-cut conclusion that, yes, primo-victimization is a major gateway to further victimization. This is actually also the case for non-autistic women: half of those who were molested as children will suffer from new occurrences of sexual violence later in life (Walker et al., 2019).

Being young made things worse in our sample: young age was a significant factor of revictimization later in life. One possible contributor to this mechanism is that children who are molested often exhibit disturbed behaviors. As Mandell et al. (2005) found out, molested autistic children were ten times more likely than the others to act out in a sexual way. Such reaction is particularly maladaptive, since hyper-sexualized behavior by children facilitates exploitation and abuse by offenders. To make things worse, it happens that autistic girls are entering puberty significantly earlier than non-autistic girls, and earlier than autistic boys as well (Corbett et al., 2020). This precocious puberty is in itself enough to put those girls even more at risk as it was demonstrated in a study by Skoog and Özdemir (2016): “early maturing girls are sexually harassed as a result of natural and normative sexual development, which happens earlier than for most of their peers.” In any case, natural early maturing and maladaptive oversexualization are extremely easy to spot, therefore attracting predators.

### Rape Severely Impacts Health of Victims

As mentioned in the introduction to this text, sexual violence has a very negative impact on health. In our sample, participants who had endured sexual violence exhibited significantly more comorbidities than participants who hadn’t. Further testing suggested that, among all comorbidities, it is specifically PTSD that is increased after the aggression, in accordance with a recent meta-analysis (Hailes et al., 2019). Given the small number of non-victims in our sample, results were not clear cut: test of equal proportions was only significant without *post hoc* correction and the *p*-value of logistic regression did not reach significance despite high odds-ratio (3.08) and a confidence interval starting above 1. This is obviously a strong limitation for between-groups comparison. Notwithstanding, in accordance with Roberts et al. (2015) and Kildahl et al. (2019), we postulate that sexual assault actually increases the risk of PTSD in autistic women, as it does in the general population. Within the victim group, however, statistics results are unambiguous. PTSD rates and RAADS scores correlate strongly, as it was described by Roberts et al. (2015), though it is not possible to determine causality. Actually, if there is no doubt that mental health in general and sexual violence victimization are strongly correlated, causality seems to be bi-directional (Machisa et al., 2017; Oram, 2019): victimization affects mental health very negatively on the one hand, and suffering from mental health problems dramatically increases the risk of victimization on the other hand. Such vicious cycle explains well the mechanism of revictimization. In our study though, being young at first aggression was a significant predictor of developing PTSD. In contrast, being victim of several aggressions doesn’t seem to increase the risk of PTSD. Therefore, it seems that for the specific case of autism and PTSD, it is really sexual aggression that induces PTSD in autistic women and not the other way around.

Besides PTSD, victims also reported consequences that occurred within the 6 months following the assault. Half of them suffered from sleep disorder (48.2%) and disgust for sex (46.7%) and third of them attempted self-harm (31.7%). It is specifically rape that induces such deleterious effects, as opposed to victims of attempted rape only or unwanted sexual touching only. Of course, this is to be interpreted with caution because there is a discrepancy in sample sizes: 44 victims did not endure rape while 155 did.

Other major vulnerability factors associated with autism and with mental health issues such as social isolation, social stigma and social rejection should be further studied in order to better describe the mechanisms of abuse (Little, 2009; Edelson, 2010).

### Few Victims Report Sexual Violence

In our study, only a third of the victims reported the assault (34.6%, *n* = 69). For the vast majority of those victims (*n* = 52, i.e., 75.4% of those who reported), such accusation had strictly no effect (no medico-legal action was taken). Even worse, 18 out of 52 were not even believed. A lot has been written about the devastating effects of denunciating sexual violence only to be ignored or even be considered a liar, adding insult to the injury. This is not at all specific to autism: an abundant literature indicates that victims of sexual violence, whatever their gender, age and social status, often experience similar skepticism and indifference to their suffering (Burt, 1980; Ahrens, 2006; Suarez and Gadalla, 2010; Kennedy and Prock, 2018; Bhuptani and Messman, 2021). Ahrens writes that “Speaking out about the assault may therefore have detrimental consequences for rape survivors as they are subjected to further trauma at the hands of the very people they turn to for help” (Ahrens, 2006). This overpowering phenomenon of disbelief has been shown to be the main reason why most victims do not talk at all about it, internalizing pain and shame instead (Kennedy and Prock, 2018). It also explains why major and coordinated social movements such as #Metoo appear to many victims as the only way toward regaining agency and dignity. Furthermore, in our study, among the very few participants (*n* = 17) whose testimony was acted upon, only 13 received care and only 12 filed a complaint. How many of those 12 complaints resulted in the perpetrators being actually condemned is here a matter of imagination as such a question was not part of the questionnaire.

### Autism Is a Risk Factor, but in What Way?

We have explored whether autistics traits, translated into metrics thanks to the RAADS scale, could be related to victimization. Results do not indicate clearly that autism is in itself a factor. Indeed, we found only one significant result: logistic regression indicated that the higher someone scores on the “sensory reactivity” sub-scores of the RAADS scale, the more elevated the risk of sexual victimization is. It is impossible to infer the direction of a putative causal effect, or even if it is just a correlation due to a confound yet to be determined. Very reactive individuals are indeed easily identified (they may wear sunglasses or noise canceling headphones) and that may help sexual predators to spot them. However, it could be the opposite: victims could display heightened reactivity due to trauma (a PTSD symptom). And last, it could be two effects of a common cause: for example, the trauma of being severely bullied at school could increase such sensitivity while at the same time making the victims more vulnerable to sexual violence.

However, a true effect of autism cannot be excluded of course. We did find a significant correlation between PTSD and total RAADS score after all, and PTSD was significantly correlated with victimization. But that could be interpreted both ways: (i) high autism traits lead to more victimization, leading to more PTSD, or (ii) PTSD might be the confound mentioned above, with PTSD being at the same time a consequence of sexual violence and the cause of heightened sensitivity in adulthood. In any case, it must be reminded here that the very small number of non-victims, the statistical negative results of our between group comparison are not very reliable. The absence of proof is not proof of absence and further research is needed here.

### What Could Prevent Sexual Victimization of Autistic Women?

At that point, it seems important to ponder about the fact that several of the publications that we have cited above mention sexual education as the primary prevention method against sexual violence. Brown-Lavoie et al. (2014) for example, assert that sex education mediates the rates of abuse^7^. However, in the case our sample, such a solution would not be deontologically applicable since half of the victims were below the age of consent, and therefore out of the scope of applied sex education. Even for victims who were aged between 15 and 18, this is not applicable: expecting minors with a disability to protect themselves thanks to education can be equated to another form of victim-blaming. Suggesting to a victim that she should have learned how to better state her personal limits is not fundamentally different than telling her that her skirt was too short. Be it in a more benevolent and well-intended way, it is as hurtful and unfair.

This is not to say that there is no need to be informed. Quite the opposite, sex education is very important for everyone in order to foster safe relationships. But, according to Sala et al. (2019) sex education programs teaching how to stay safe from abuse are poorly validated. More importantly, considering that it is the victim’s duty to prevent the sex crime is without doubt a remnant of rape culture^8^. Regrettably, the opposite proposition is not as often suggested. Educating potential offenders instead of educating potential victims would be more ethical and might prove a more efficient method, although it would be of limited efficiency since offenders are usually not enamored youngsters unable to control their sexual urges, thereby becoming unintentional sex offenders. That is simply another rape myth (Payne et al., 1999). On the contrary, most offenders are smart predators, who are very aware of what they are doing (Burt, 1980; Hanson and Morton-Bourgon, 2005; Methot-Jones et al., 2019; Cardona et al., 2020; Russell and King, 2020). As Russell and King (2020) describe it: “Psychopathy and narcissism are known predictors of sexual violence.” Moreover, the callousness and cynicism of perpetrators is actively supported by a systemic rape culture, that is a “sociocultural context that sexually objectifies the female body and equates a woman’s worth with her body’s appearance and sexual function,” an internalized mechanic called objectification theory (Szymanski et al., 2011). As Methot-Jones et al. (2019) write, “individuals high in psychopathic traits see women as sub-human, this dehumanizing appraisal may be facilitating attitudes and behaviors that are consistent with the idea that women are less than human and deserve to be treated as such.” Objectification theory is particularly well illustrated by the systematic use of rape as a weapon of war in all ages and cultures (Bourke, 2014).

It seems intuitively right that, since autistic girls may misunderstand the complex games of flirting and dating, teaching them on how to behave in romantic relationships would help them achieve a satisfying love life (McMahon et al., 2021). However, this can only be successful in equal, fair and respectful relationships, not in situations where they are preyed upon. As Gotby et al. (2018) wrote in their study: “our findings that there were no specific effect of ASD or ADHD symptoms when controlling for general symptom load, fail to lend support to the hypotheses that social deficits, communication difficulties or impulsivity […] mediates the association between ASD/ADHD and coercive sexual victimization. This suggests that rather than focusing on a specific symptom-cluster or diagnosis, it is the general [neurodevelopmental disorder] phenotype that predicts increased risk of victimization” (Gotby et al., 2018). This is in agreement with research showing that women with ADHD are also at very high risk of being victimized (Snyder, 2015). It means that it is not autism in itself, nor the social communication difficulties associated with autism, that increase vulnerability to sexual violence but the fact of being perceptibly different. Or, alternatively, being different increases the risk of bullying and rejection, and in that case, it would be ostracization and stigma that would be the actual factors increasing victimization. Of course, as stated by Hartmann et al. (2019) sex education may still prove to be marginally protective: “While individuals with ASD are certainly not at fault or responsible for experiences of sexual abuse or victimization, misinformation about healthy sexuality (e.g., personal space, communication of consent, appropriate and inappropriate sexual behaviors) may in some cases contribute to adverse sexual encounters” (Hartmann et al., 2019).

Beside those considerations, another major criticism against the “preventing through sex educating” method is that victims are very often in a state of stupor and dissociation during the assault. They are therefore unable to react, sometimes even unable to comprehend, and no education can prevent that. Let’s remember here that the overwhelming majority of sex crimes are committed by persons who are closely related to the victims, a parent, a teacher, a boyfriend (WHO, 2021; Sardinha et al., 2022). In some cases, the state of psychological shock that accompanies such assault by a loved one can be so massively devastating that the victim is stunned into derealization and cannot understand, or even remember, what is happening. As one of our participants wrote: “There was no use of force, no threats, nothing. It was in the waiting line to the cafeteria. I was frozen.”

The SES-SFV responses provided yet another counterargument to the model of sex education as a prevention tool in that it collects information about the strategies used by offenders. Perpetrators primarily used two strategies to violate and abuse: manipulation/mental ascendency/harassment (“a”) and using surprise to take advantage (“c”). Strategy “c” was the first choice for imposing unwanted sexual contact, while strategy “a” was preferably used for rape attempt and rape. This might prove crucial for understanding why and how sexual violence happens so often in this population. As we have seen, one out of three non-autistic women is a victim of sexual violence. How could autistic women who, by very definition, are ingenuous and cannot guess hidden motives, defend themselves better than non-autistic persons against deviousness, treachery and deception? As Roberts et al. (2015) accurately write: “Deficits in emotional and social cognition, specifically, inability to identify sexually inappropriate behavior […] and inability to identify one’s own discomfort at inappropriate behavior […] increase risk of victimization and characterize persons with autistic traits.”. One participant wrote “It was because I believed that he would love me. I was raped and I never had any other experience since then. I was 19, I am now 36.” Another participant stated that she had “sexual relationships before the age of 15 with an adult over 30 who clearly took advantage of the situation.”

Interrogated about prevention methods, one victim out of ten answered that nothing could have protected them. One out of four considered that their autistic traits made them easy to spot by sexual predators. Four out of ten thought that knowledge about risk and self-affirmation strategies could have prevented the assault. However, as we asserted, the high proportion of underage victims renders such a method irrelevant since it would mean putting the responsibility of avoiding victimization on the shoulders of the minor victims.

In addition, it is very important to remember that, contrary to widespread myths regarding rape, offense is almost always perpetrated by a close person, exploiting ascendancy or hierarchical power over the victim. What could education do against situations such as the ones reported by our participants: “I was repeatedly raped by a relative during infancy and childhood”; “the first time, I was 6 years old”; “someone who had authority over me (father)”? On the contrary, as mentioned by one out of five participants, the most efficient protection, especially in the case of vulnerable individuals, is the presence of a vigilant caregiver. Educating families and professional about the risk of sexual victimization of girls on the autism spectrum should therefore be a priority.

Our research indicates that almost 9 autistic women out of 10 are sexually victimized, at huge costs for their mental and physical health. Of course, one major limitation of our study is that it is based on voluntary participation and that represents a bias since concerned individuals, i.e., victims, may be more willing to contribute to such research than non-victims. However, this bias might not impact results a lot, since our findings are very consistent with literature about sexual violence against autistic women and against women in general. So, what could be done to prevent such generalized issue? The WHO study (WHO, 2021) shows unambiguously that sexual violence is systemic and that vulnerable individuals are preferably targeted by offenders. Therefore, we state that it would be a methodological and deontological mistake to consider that victimization in autistic women is mainly due to autism. On the contrary, we postulate that the main cause of sexual violence against autistic women is their womanhood, since perpetrators preferably target women and girls. Therefore, it is reasonable to consider that autism is not the cause of sexual victimization in autistic women but just a factor increasing their vulnerability.

Prevention is absolutely necessary, certainly not in the sole form of sex education but rather by promoting profound cultural changes such as recommended by the Center for Disease Control (CDC) and World Health Organization (WHO): both organizations indeed strongly affirm that the very root of sexual violence is gender inequality. As Jessica Leight formulates it (Leight, 2022), “the literature analyzing the effectiveness of strategies to prevent and reduce intimate partner violence […] is now encapsulated in the RESPECT framework and its implementation plan.” As a tentative conclusion, we report here the most recent recommendations for preventing sexual violence as published by the CDC and the WHO. From the CDC, the Division of Violence Prevention of the National Center for Injury Prevention and Control has published a technical package to prevent sexual violence, titled the STOP-SV (Basile et al., 2016), that advocates sexual violence prevention along five dimensions:

• S: Promote **Social Norms** that Protect Against Violence
• T: **Teach Skills** to Prevent Sexual Violence
• O: Provide **Opportunities to Empower** and Support Girls and Women
• P: Create **Protective Environments**
• SV: **Support Victims/Survivors** to Lessen Harms

The WHO has published a framework, very consistent with the above model, titled the RESPECT framework (World Health Organization, 2019).

• R – **Relationship skills strengthened**. This refers to strategies to improve skills in interpersonal communication, conflict management and shared decision-making.

• E – **Empowerment of women**. This refers to economic and social empowerment strategies including those that build skills in self-efficacy, assertiveness, negotiation, and self-confidence.
• S – **Services ensured**. This refers to a range of services including health, police, legal, and social services for survivors of violence.
• P – **Poverty reduced**. This refers to strategies targeted to women or the household, whose primary aim is to alleviate poverty.
• E – **Environments made safe**. This refers to efforts to create safe schools, public spaces and work environments, among others.
• C – **Child and adolescent abuse prevented**. This includes strategies that establish nurturing family relationships.
• T – **Transformed attitudes, beliefs and norms**. This refers to strategies that challenge harmful gender attitudes, beliefs, norms and stereotypes.

In her seminal article, “Cultural myths and supports for rape,” Martha R. Burt wrote that “the task of preventing rape is tantamount to revamping a significant proportion of our societal values” (Burt, 1980). The CDC and WHO sexual violence prevention programs, designed four decades after Burt wrote those words, indicate that such profound change is taking place. Vulnerable populations, such as autistic women, shall benefit from this evolution.

## Data Availability Statement

The datasets presented in this study can be found in online repositories. The names of the repository/repositories and accession number(s) can be found below: Repository URL for dataset is: https://nakala.fr/10.34847/nkl.d3d35i2o; Repository URL for R code is: https://nakala.fr/10.34847/nkl.4021h29a.

## Ethics Statement

Ethical review and approval was not required for the study on human participants in accordance with the local legislation and institutional requirements. The patients/participants provided their written informed consent to participate in this study.

## Author Contributions

FC: writing and supervising statistical analyses. ER: statistical analyses and proofreading. SL: co-initiator of the study and experimental design. DG: initiator of the study, experimental design, data collection, and preliminary analyses. All authors contributed to the article and approved the submitted version.

## Conflict of Interest

ER was employed by Auticonsult. The remaining authors declare that the research was conducted in the absence of any commercial or financial relationships that could be construed as a potential conflict of interest.

## Publisher’s Note

All claims expressed in this article are solely those of the authors and do not necessarily represent those of their affiliated organizations, or those of the publisher, the editors and the reviewers. Any product that may be evaluated in this article, or claim that may be made by its manufacturer, is not guaranteed or endorsed by the publisher.

## Footnotes

1 Unless otherwise specified, “woman” is equivalent to “assigned female at birth” in this article.

2
https://www.who.int/reproductivehealth/topics/violence/sexual_violence/en/

3 LGBTQIA++: lesbian, gay, bisexual, transgender, questioning, queer, intersex, asexual, pansexual, and allies. In this text, we chose to use the umbrella term “queer.”

4
www.surveymonkey.com

5 As mentioned above, the details can be found in the commented version of our R code (Repository URL for R code is: https://nakala.fr/10.34847/nkl.4021h29a).

6 “High functioning” here means that our participants had to be able to fill a questionnaire in order to be included.

7 We are doubtful of Brown-Lavoie conclusion because the sex education they assessed was about STD and birth control, not about being safe from abuse. It is very possible, however, that sex education as they defined it was a confound.

8 Cf. the Gatekeeper model.
