# Supplementary material for: Evidence That Nine Autistic Women Out of Ten Have Been Victims of Sexual Violence
Source: Front Behav Neurosci. 2022 Apr 26;16:852203. doi: 10.3389/fnbeh.2022.852203 (PMC9087551; doi:10.3389/fnbeh.2022.852203)
Supplement: Supplementary file 1 [file Data_Sheet_1.pdf]

SUPPLEMENTARY MATERIAL: methods section

Interactions between assault types

Conditional probabilities, chi-square tests, McNemar tests and two-sample tests for equality of proportions were run to test whether or not having undergone a given type of assault made a participant more likely to have undergone another type of assault. The condprob() function of the bindata R package allowed to run conditional probabilities on a table consisting of only the binary versions of the sexual touching victimization column, the attempted rape victimization column, the overall rape victimization column, the oral rape victimization column and the vaginal/anal rape victimization column. Chi-square tests were run separately on all assault type columns and all question answer columns. Making 2x2 contingency tables for columns by pair allowed for both chi-square tests and McNemar tests. **In the two-sample tests for equality of proportions, the full sample was divided into the victims and non-victims of a given type of assault. Being the victim of another type of assault was then treated as a "success" for each group.** The results of the test are presented in table [x].

New binary columns, with victims coded as 1 and non-victims as 0, were created to distinguish the participants that had answered positively to only one of the questions, two questions, three or all of them. Different columns were made for each single question, possible combination of two questions and possible combination of three questions. The same was done for types of assault, with distinct columns for each single type and combination of two. A new binary column in which non-victims for all questions and all types of assault were coded as 1 instead of 0 was also made. The binaries on the questions allowed us to get the percentage of participants who had answered positively to all combinations of questions. A table made of only the new binaries for type of assault and the new non-victims binary was reshaped into a table that contained two columns: one with the assault combination undergone, the other a binary column, with each assault combination taking up as many rows as the number of participants. All rows in which the binary was 0 were removed. The resulting table was used to make figure [x].

Distribution of assaulter strategies

The data for the strategy used during assaults was retrieved from Survey Monkey in the form of four columns, each corresponding to a question from our modified version of SES-FV. Each of the columns contained one answer per participant, which could be strategies a-e or declaring oneself to not be a victim. A table made of only those four columns was reshaped into a table that contained two columns: one with the question that was being answered and one with the answer that was given, each question taking up as many rows as the number of participants. All rows in which the answer was the "non-victim" option were removed. At that stage, it was possible to get the percentage of victims that had encountered each strategy for each question.

To get the numbers per type of assault, we renamed the questions in such a way that any answers associated with the first question of our modified SES-FV were associated with sexual touching, the answers to both the second and third question were associated with rape and the answers to the fourth were associated with attempted rapes. Because of this change, the "rape" category could no longer be guaranteed to contain only one answer per participant, so the distribution of strategies in each assault is considered a percentage of answers rather than a percentage of individual victims

who encountered that strategy. This table was the basis for figure [x].

Chi-square tests were done on a contingency table of all five strategies and all types of assault to see if there were any differences in the distribution of strategies between the three types of assault. The "d" strategy turned out to be completely absent from answers related to sexual touching and statistically quasi-absent from both attempted rapes and rapes. We used two correction methods available: simulating the p-value via an option available in R and re-doing the chi-square test on a version of the table from which all rows containing the "d" strategy were removed.

We next looked at how the proportions of strategies, "a", "b", "c" and "e" differed individually between assaults, not doing so for "d" because its frequency had already been shown to be too low for reliable testing. Four copies of the two-column table were made. In the first, the strategy column was modified in such a way that each row containing the "a" strategy became a 1 and each row containing strategies "b", "c", "d" and "e" became a 0. The three other copies of the table underwent the same process, except that the strategy turned to 1 was "b", "c" and "e" respectively. Those four tables were all turned into contingency tables to which a chi-square test was applied to see if the difference remained on a divide between only one strategy and all the others. Next, the rows corresponding to rape were removed from the table to do a chi-square that compared only the proportion of any given strategy between sexual touching and attempted rapes. Via repeating this process with the two other types of assault, we were able to compare the differences between sexual touching and rape, then between rape and attempted rape. The results we got added Yate's continuity correction. Since some the p-values we got were between 0.10 and 0.05 after the correction, we re-did the chi-square test without the correction to see if any of them dipped under 0.05 upon its removal.

Processing SES-FV results

The recommended analysis method for answers to SES-FV requires one to first look at both questions and answers to translate them into one of six situations: non-victim, sexual contact, attempted coercion, coercion, attempted rape and rape. The divide between sexual contact, an attempted rape and rape is reliant on the question answered, while the divide between coercion and an assault is based on the strategy that was used. Even in our simplified version of the questionnaire, all six of those situations are possible, as we still have a question for sexual contact, two questions for rape and one for attempted rape. However, we do not consider the distinction between coercion and assault to be relevant to our study, which results in the questions about rape and attempted rape being treated much like the one about sexual contact in the recommended method: a participant is a victim of the act regardless of the strategy used. This results in our data now having only four levels of sexual assault seriousness: 1: Non-victim; 2: Sexual touching (sexual contact renamed to avoid getting it mixed up with the more broadly defined sexual assault); 3: Attempted rape; 4: Rape. The creation of new columns in which the conversion from strategy-based answers to this hierarchy was one of the very few transformations done in the Excel file proper.

We also looked at the results under angles other than just the "most serious" act undergone by each participant, as to not let cases of rape overshadow "less serious" acts undergone by each participant. In addition to the maximum score for each participant, we made analyses on the columns we had at our disposal to make victim(1)/non-victim(0) binary columns for future analyses, including one that accounted only for both types of rape. We also created a function that allowed us to know what our total of victims would be if rape was ignored as a potential act and attempted rape was the most serious act asked about, with sexual touching included; the next step down is the same thing as the

hierarchy version of the answers to the sole question about sexual touching. Any future mentions of the questions will refer to the question about sexual touching as s1, those about rape as s2 (oral) and s3 (vaginal/anal), and the one about attempted rape as s4.

Overlap checks on the data

We made a few contingency tables to check for some overlaps between the open question answers and SES answers. We looked at how many participants who had chosen at least one of the positive answer columns (the assault, rape and attempted rape options) in the open question were also victims according to SES and how many people who hadn't were also non-victims according to SES. We looked at how many participants who didn't know in the open question were victims according to SES, and how many weren't. We also looked at how many people who had declared never having undergone a sexual assault in the open question were non-victims or victims according to SES.

We also checked how the RAADS scores of self-diagnosed participants and victims according to SES were distributed compared the RAADS scores of all participants, as to check for any trend among those sub-populations. Those distributions can be examined in figure x and figure x.

Early data cleaning

The participants were informed that the survey was intended to become a published study. At the end of the survey, they were allowed to choose whether to be part of the study or not. The first potential participants we excluded were thus those who had given a negative answer to that question.

Our second criterion for excluding potential participants from the start was the RAADS score. We used a total calculated directly in Excel. The scores to individual questions came with a few problems. One was that SurveyMonkey returned numbers 1-4 in each column, when the scale for individual RAADS questions is 0-3. In addition to this, for the fifth question (out of 14), some participants had apparently been allowed an "other (please specify)" option, in which case the number returned by SurveyMonkey was 0. To circumvent this, we first decided to not count such cases as part of the overall RAADS score. Second, after summing up each line, we subtracted the number of columns as an equivalent to removing 1 from each number added up, making sure to skip the fifth question's column when needed. We also had to account for the fact that the scale internal to individual columns needed to be reversed for the sixth question. We used the total RAADS score calculated from those operations to filter out the people whose total score was under 14, which is the threshold at which people are considered to be on the autistic spectrum.

As the survey didn't contain any means to allow parental approval in place of a minor that we knew of, we also removed all participants who put their current age in the "17 years old or younger" category.

Processing the open question results

The open question was presented in such way that it was possible to choose several answers. The possible answers were to claim to have never undergone a sexual assault, to have undergone one or several sexual assaults, to have undergone a rape, to have undergone an attempted rape, to not know, or something else that could be written in. The form in which the data was obtained from SurveyMonkey was in the form of six columns in which the cell was completely empty if the participant in a given row hadn't chosen the answer. If the participant had answered, the cell

contained a number for the five first questions and the text of the write-in for the sixth. Binaries for individual columns were made, as with SES. A method was developed as an alternative divide on which to do tests looking for differences between victims and non-victims as defined by SES. It separated participants that been able to affirm that they had undergone an assault, a rape or rape attempt as soon as the open question from the rest of the participants.

Comparing answers from the two sexual assault questionnaires

We used the binaries from SES and the open question to compare the answers to similar questions for the two questionnaires. The answers about assault from the open question were paired with the question about sexual touching from SES, the answers about rape from the open question were paired with the combined rape column from SES and the two columns about attempted rape were paired with each other. Contingency tables were made for each pair. McNemar and Chi-square tests were run on the contingency tables. The numbers were used for figure [x]. We also looked at the answers given to SES by participants who had declared not knowing if they had been sexually assaulted or, more rarely, declared not having been assaulted in the open question.

Processing the RAADS score

We calculated the mean, median and standard deviation of the RAADS scores across our sample of participants from the total that had already been calculated. We collected the individual RAADS columns into their own table, using the inverted version of the column for the sixth one. Write-in answers to the fifth RAADS column were scored 0 on the 1-4 scale used in the SurveyMonkey data. We changed each 0 to a 1 so that, when we next removed 1 from the value of all scores in the table, the influence of the former 0 scores remained neutral. The individual RAADS columns were next used to calculate three sub-components of RAADS for each participant. The “social communication” component is the sum of the scores from columns 1, 4-6, 8, 9 and 11. The “hyper-focalization” component is the sum of columns 12-14. The “sensory reactivity” component is made of the sums of columns 7 and 10.

Estimating disorder numbers

The data for the presence of disorders other than autistic spectrum disorders came from SurveyMonkey the same format as the answers to the open question about assault. This data was used to make binaries for the presence or absence of each disorder in each participant. Next, the sum of all possible answers for the presence of disorders, except for the options to declare having none of those suggested and the write-in-answers, was made for each participant. Binaries based on the SES answers and the open question answers were used to get the mean, median and standard deviation of several pairs of subgroups and the worse act hierarchy from SES. Those pairs of subgroups were then compared to each other with a Wilcox test, while Kruskal-Wallis was used on the four subgroups contained in the worse act hierarchy. This allowed us to know whether some subgroups tended to have a higher number of other disorders than others.

Wilcox tests and logistic regressions: victimization function of RAADS score

The mean, median and standard deviation of the RAADS score and each of its components were calculated for victims and non-victims via three different divides. There was as divide based on assault victims and non-victims per SES, and another based on definite victims and the rest per the open question. Wilcox tests were made to see if the distribution of the RAADS score or any of its

components changed between victim and non-victim groups. We also made several logistic regressions in which the dependent variable was one of the two victimization scenarios above and the independent variable was the RAADS score or one of its components.

Logistic regression on presence of PTSD

We made logistic regressions in which the dependent variable was the presence or absence of PTSD. The first independent variable we tested was having been sexually assaulted. We also tested age at first assault alone and total RAADS score alone as independent variables. We also created figure [x], a graph separating participants by their age at first assault that shows which percentage of each group has PTSD.

Part 2 of treating "count-only" variables

Sex, current age, professional status, marital status, education level, health status, iq, sexual orientation, past sexual relationships, regularity of sex over the past six months, age at first assault, whether the assault had been spoken about or not, and prevention methods that could have helped were all single-choice questions, resulting in them being in one column each. Those columns were all processed together to get the needed numbers. The origin of the diagnosis and the question asking whether there had been one or several sexual assaults were multiple choice, resulting in the number of participants that gave each answer being separately processed in each case.

Operations done of the number of assaults

The question about the number of assaults had four possible answers, which were no assault, only one assault, several assaults from a single person or multiple assaulters. The columns containing those answers were changed into binaries. Figure [x] separates all the participants by age at first assault and shows the number of people who underwent assaults from several people.
We did a logistic regression in which having had several abusers was the dependent variable and the age of first assault the independent variable. Another logistic regression used having undergone several assaults from the same person as the dependent variable to check for any similar trends. Among assault victims per SES, two separate logistic regressions were used to check if having several assaulters or undergone several assaults from the same person had any predictive power for to the presence of PTSD. Two-sample tests for equality of proportions, with the group divide based on the existence of several assaulters and "success" being the presence of PTSD were also done with the purpose of answering the same question.

Presence of other disorders

The number of participants presenting non-AS disorders was recorded for the full sample, for victims and non-victims of assault, and for victims and non-victims of rape. Both victim and non-victim divides were used, with the number of rape victims according to the one based on the open question being the number of participants who had declared having been raped. Two-sample tests for equality of proportions, with the sample divided along victim/non-victim lines and presence of the disorder as the "success" were done for sexual assault and rape. A third combination of groups was made out of rape victims and sexual assault non-victims. As two-sample tests for equality of proportions stop working when the number of expected successes in one of the groups gets too small, only the four most frequent disorders were tested: depression, anxiety, PTSD and ADHD, with the latter's numbers among non-victims sometimes being too low for test results to be reliable.

Consequences within six months of the assault

The assault's consequences for the victim were part of a multiple-choice question in which all but one were intend for assault victims. Before counting the number of people for which any given consequence happened, we made sure to cut assault non-victims out of the table in one instance and rape non-victims in a second instance. Figure [x] was made with the data from all possible answers except the write-in option. Participants in each consequence are separated by the worse act they have undergone, with a no assault > sexual touching > attempted rape > rape hierarchy. The "nothing happened" option was kept because the distribution of situations inside it hint that it was misunderstood by some participants as a "underwent an assault, but there were none of the consequences listed here" option rather than the "nothing because there was no assault" option it was intended to be.

Overlap of non-victim definitions

The questionnaire gave the participants a total of seven occasions to declare that they weren't a victim of sexual assault: declaring not having undergone an assault during the open question, not giving a strategy in SES, not declaring an age of first assault, declaring no assaults in the question about the number of assaults undergone, declaring there was no assault to talk about, declaring oneself not affected when asked about prevention and declaring that nothing happened when asked about consequences. A column resulting from processing SES and six other columns were assembled in their own table and all transformed as to highlight the non-victims, rather than victims to check for overlap of non-victims of all questions. Here, non-victims were coded as 1 and victims as 0.

We added a column consisting of the sum of each row, then did a transformation to see how many times a given sum appeared. A surprisingly low number was found both for participants who qualified for all definitions and for the number of participants who qualified for all seven. Participants who qualified for only one, two, three, four, five or six definitions according to their answers were found. In the light of this, we used the condprob function from the bindata package on the table to see how often given definitions were found among the same participants. There was also a lead in starting to explain those results that seemed solid enough to test and record the results of: the number of non-victims via only one definition for whom said sole definition was the oft-misunderstood "non-victim" option for the consequences within six months of the assault.
