# Supplementary material for: Evidence That Nine Autistic Women Out of Ten Have Been Victims of Sexual Violence
Source: Front Behav Neurosci. 2022 Apr 26;16:852203. doi: 10.3389/fnbeh.2022.852203 (PMC9087551; doi:10.3389/fnbeh.2022.852203)
Supplement: Supplementary file 2 [file Data_Sheet_2.pdf]

English translation of the survey questions from [Article final title]

(Because of quirks of the French language, the verb “experiencing” was the most appropriate translation for a verb used in sentences that also contain “experience” as a noun)

Asperger Syndrome is a form of autistic spectrum disorder that characterizes itself by difficulties in social interaction in people of high intellectual level. Research is today more interested in the profile of women with Asperger’s Syndrome (“Aspergirls”), who are characterized by their great emotional sensitivity, their capacity to integrate better than Asperger men, but also their great sexual vulnerability. However, there is currently only very few scientific data on problems specific to the sexuality of these women. By participating in this research:

- You will contribute to allowing to better understand, prevent and cure problems relative to sexuality in “Aspergirls”. Thank you for your participation!
- This questionnaire is strictly anonymous, IP data is not tracked (please only fill this survey once per person). The CNIL declaration n° is 2172655.

1) What is your sex?

2) Have you been diagnosed by a psychiatrist as coming under the Asperger Syndrome diagnosis?

3) What age are you?

4) Among the following categories, which one best describes your current professional status?

5) What is your marital status?

6) What is the highest education level that you have reached?

7) Generally, how would you assess the global state of your health?

8) The diagnosis of you Asperger’s syndrome was done:

- In an expert center?
- By a child psychiatrist during your childhood?
- By a psychiatrist when you were an adult?
- By a general practitioner?
- Was the assessment completed by an assessment made by a specialized neuropsychologist?
- Did you do your own diagnosis without getting confirmation from a psychiatrist?
- Other (please elaborate)

9) If you have gotten a test evaluating your intellectual efficiency (WAIS - Wechsler Adult Intelligence Scale - or WISC -Wechsler Intelligence Scale for Children- type IQ test), done by a psychologist, what was your result? NB: If it was not possible to calculate the global IQ, take the highest number (f ex if verbal IQ is 115 and performance IQ is 90, take 115)

10) I feel sexually attracted by:

11) Over the course of my life, I have had relationships with:

12) Over the last six months: (answers involve the frequency of sex)

13) Do you think you have been the victim of sexual abuse?

- No, never
- I underwent one of several acts of sexual abuse
- I was raped
- I underwent an attempted rape
- I don’t know
- Other (please elaborate)

The following questions come from the RAADS-14 questionnaire (Eriksson et al., 2013) whose purpose is to briefly evaluate the main features that can be found in people with Asperger Syndrome. Answer spontaneously. There is no right or wrong answer, it is meant to get a better picture of your personality.

14) RAADS)1 - It’s often difficult for me to understand how people feel when we speak with each other.

15) RAADS)2 - Some ordinary textures that don’t bother others feel very unpleasant when they touch my skin.

16) RAADS)3 - It’s very difficult for me to work and function in groups.

17) RAADS)4 - It’s often difficult to understand what others expect of me.

18) RAADS)5 - I often do not know how to act in social situations.

19) RAADS)6 - I can talk and converse with people.

20) RAADS)7 - When I feel submerged by my senses, I need to isolate myself to calm myself down.

21) RAADS)8 - How to make friends and socialize is a mystery for me.

22) RAADS)9 - When I’m talking to someone, I have trouble telling when it’s my turn to speak or listen.

23) RAADS)10 - Sometimes, I need to plug my ears to block out painful noises (like the sound of a vacuum cleaner or people talking too loudly).

24) RAADS)11 - It can be very difficult to read face, hand and body movements of someone when we talk.

25) RAADS)12 - I focus on details rather than general idea.

26) RAADS)13 - I take thing to the letter too much, which results in me often missing what people are trying to say.

27) RAADS)14 - I become very upset when the way I like to do things is suddenly changed.

The questions you are about to read are extremely precise because the researchers (SES questionnaire: Sexual Experiences Survey; Koss & al., 1987) who built it voluntary wanted to eliminate all ambiguity in the wording of those questions. Those questions can make you uneasy. If you feel too anxious while taking the test, feel very free to stop and don’t hesitate to contact your doctor to talk about it.

28) S.E.S-1) Someone has petted, kissed, or rubbed themself against intimate parts of my body (lips, breasts, pubis, buttocks) or taken some of clothes off without my consent.

29) S.E.S-2) Someone has had oral sexual relations with me or made me have oral sexual relations with others without my consent.

30) S.E.S-3) Someone has inserted their fingers, their penis or an object in my vagina or anus without my consent.

31) S.E.S-4) Someone tried to have sexual relations, or to make me have sexual relations with others without my consent, but didn't succeed.

32) If you have answered something else than never to one of the four previous questions from the S.E.S questionnaire, it's possible that you have been a victim of sexual abuse. It's very important to talk about it to your psychiatrist or psychologist so they can advise you and help you. What age were you the first time you experienced one of those non-desired sexual experiences?

33) If you have experienced or suspect having experienced a non-desired sexual experience: (answers involve number of assaults)

34) If you have experienced (or suspect having experienced) a non-desired sexual experience and that you told someone about it, at the time, what happened?

35) If you have experienced a non-desired sexual experience, do you consider that (choose the option that seems the most important to you in your case): (answers involve prevention)

36) Are you or have you been in a psychiatrist's care for one of the following disorders?

- Depression
- Attention deficit hyperactivity disorder (ADHD)
- Borderline personality disorder
- Bipolar disorder
- Anxiety disorder
- Alcohol problems
- Drug problems
- State of post-traumatic stress
- Schizophrenia
- None of those problems
- Other (please elaborate)

37) If you have experienced one of those non-desired sexual experiences, what have the consequences been for you in the six months that followed the abuse?

- Suicide attempt(s)
- Act(s) of self-harm
- Alcohol and drug abuse
- Tattoo(s) and/or piercing(s)
- Sleep trouble and repeated nightmares
- Deep disgust for sex
- Significant weight gain (+10% of your base weight, not attributable to a psychotropic)

- Beginning of a psychiatrist’s care
- Prescription of psychotropics
- Not applicable (I didn’t experience such an experience)
- Other (please elaborate)

38) The questionnaire is finished, and we thank you. The results of your questionnaire are not going to be processed individually but collectively with the results of other patients (we wish to collect at least 200 questionnaires) in order to produce analyses that may become the subject of a scientific publication. The questionnaire is anonymous, the IP addresses are not kept ; you will hence not be able to have access to your individual results. Do you accept that the data that you have just entered be incorporated into the ASPERGIRL-SEXP survey?
